# TRIB2 supports a high glycolytic phenotype in melanoma cells

**DOI:** 10.1371/journal.pone.0360503

**Published:** 2026-11-12

**Authors:** Victor Mayoral-Varo, Ana Luísa De Sousa-Coelho, Carla Lopes, Óscar H. Martínez-Costa, Carlos Amenabar, Paulo J. Oliveira, Juan J. Aragón, Bibiana I. Ferreira, Wolfgang Link

**Affiliations:** 1 Sols-Morreale Biomedical Research Institute (IIBM), Spanish National Research Council (CSIC), Universidad Autónoma de Madrid (UAM), Madrid, Spain; 2 University Center for Health Sciences – HM Hospitals (CUHMED), Camilo Jose Cela University, Madrid, Spain; 3 HM Hospitals Health Research Institute, Madrid, Spain; 4 Algarve Biomedical Center Research Institute (ABC-Ri), Campus de Gambelas, Faro, Portugal; 5 Escola Superior de Saúde (ESS), Universidade do Algarve, Campus de Gambelas, Faro, Portugal; 6 Center for Neuroscience and Cell Biology (CNC), Centre for Innovative Biomedicine and Biotechnology (CIBB), University of Coimbra, Coimbra, Portugal; 7 Department of Biochemistry, Faculty of Medicine, Universidad Autónoma de Madrid (UAM), Madrid, Spain; 8 Faculdade de Medicina e Ciências Biomédicas (FMCB), Universidade do Algarve, Campus de Gambelas, Faro, Portugal; Weill Cornell Medicine, UNITED STATES OF AMERICA

## Abstract

**Background:**

Metabolic adaptation is a crucial driver of melanoma progression and therapy resistance. The pseudokinase Tribbles homolog 2 (TRIB2) is highly expressed in melanoma and is associated with poor outcomes and treatment resistance. However, its specific role in the metabolic profile of melanoma cells remained unexplored. This study aimed to investigate the impact of TRIB2 on cellular metabolism, and its role in metabolic adaptation in melanoma.

**Materials and methods:**

The study used UACC-62 human melanoma cells with high endogenous TRIB2 expression and isogenic TRIB2-knockout (KO) clones generated via CRISPR-Cas9. Cell proliferation was assessed using trypan blue exclusion assays under treatment with metabolic drugs including 2-deoxyglucose (2-DG), and dichloroacetate (DCA). Metabolic phenotypes were evaluated by measuring glucose consumption, lactate production, and real-time analysis of glycolytic flux (ECAR) and mitochondrial respiration (OCR) using a Seahorse bioanalyzer. Reactive oxygen species (ROS) levels were measured by dihydroethidium (DHE) fluorescence. Gene expression was analyzed by qPCR, and protein levels were determined by western blot. Citrate synthase activity was measured enzymatically.

**Results:**

TRIB2-KO cells exhibited significantly reduced basal proliferation compared with wild-type (WT) cells. KO cells were also more sensitive to the glycolytic inhibitor 2-DG. Conversely, WT cells were more susceptible to DCA treatment. Metabolic analyses confirmed that TRIB2-KO cells had lower glucose consumption, lactate production, and glycolytic capacity than WT cells. Mitochondrial respiratory function and the protein levels of key respiratory complexes were unchanged. Despite the reduction in glycolytic activity, TRIB2 depletion did not increase mitochondrial respiratory capacity and was associated with elevated basal intracellular superoxide levels. Among the molecular alterations identified, PDK4 was consistently reduced at both the mRNA and protein levels, whereas citrate synthase activity was significantly decreased despite preserved protein abundance, indicating selective alterations in mitochondrial carbon metabolism.

**Conclusions:**

TRIB2 expression promotes a glycolytic phenotype and contributes to metabolic adaptation in melanoma. Loss of TRIB2 does not induce a metabolic shift towards oxidative phosphorylation but instead results in broader metabolic rewiring characterized by reduced glycolysis together with selective alterations in mitochondrial carbon metabolism. These findings identify the TRIB2–PDK4 axis as a previously unrecognized component of metabolic regulation in melanoma and provide a rationale for further investigating its therapeutic potential.

## Introduction

Dysregulation of cellular metabolism is a hallmark of cancer [1]. Metabolic reprogramming enables melanoma cells to adapt to environmental and therapeutic stress, contributing to tumor progression and treatment resistance [2]. Melanoma, the most lethal type of skin cancer, has witnessed remarkable advances in therapy, notably mitogen-activated protein kinases (MAPK) and immune checkpoint inhibitors. However, the potential of these approaches is curtailed by drug resistance [3,4], accentuating the need for innovative melanoma treatments. Consequently, identifying the molecular mechanisms underlying metabolic adaptation may uncover novel therapeutic vulnerabilities in melanoma.

Together with TRIB1 and TRIB3, Tribbles homolog 2 (TRIB2) belongs to the well-conserved mammalian Tribbles family of pseudokinase proteins [5]. There is evidence that TRIB2 overexpressing tumors show a malignant/chemoresistant phenotype in several types of cancer, spanning acute myeloid leukemia [6], chronic myelogenous leukemia [7], small cell lung cancer [8], glioblastoma (GBM) [9], colorectal cancer (CRC) [10,11], laryngeal squamous cell carcinoma [12], pancreatic cancer [13], and prostate cancer [14], suggesting TRIB2 as a therapeutic target for resistant tumors. In melanoma, we have identified TRIB2 as an oncogene and as a repressor of the metabolic master regulators forkhead box class O (FOXO) transcription factors through the activation of AKT [15]. Moreover, TRIB2 depletion in melanoma cells disrupts AKT/FOXO signaling in response to diverse drugs [16]. Therefore, we propose TRIB2 as a valuable biomarker for melanoma diagnose, progression assessment, and prediction of clinical responses to cancer treatments [17]. Despite the growing evidence supporting an oncogenic role for TRIB2, its contribution to metabolic regulation remains poorly understood. To date, only one study has directly linked TRIB2 to cancer metabolism, demonstrating that TRIB2 interacts with pyruvate kinase M2 (PKM2) to promote aerobic glycolysis in lung cancer cells, resulting in increased glucose uptake and lactate production [18]. Whether TRIB2 exerts a similar metabolic function in melanoma remains unknown. The aim of this study was to shed light on the role of TRIB2 in the metabolic phenotype of melanoma cells.

## Materials and methods

### Cell lines and treatments

Isogenic melanoma cell lines, corresponding to WT [TRIB2(+)], and CRISPR/TRIB2-KO [TRIB2(-), undetectable TRIB2 protein, clones #28 and #31] UACC-62 cells were developed and described previously [16]. Briefly, after cells were transfected with two independent guide RNAs in plasmid vectors expressing Cas9 nuclease protein, single-cell clones were selected and expanded. Finally, the full deletion of the protein levels of TRIB2 were confirmed by Western blot.

Cells were cultured in standard conditions of temperature (37°C) and CO_2_ (5%), and maintained in DMEM high glucose (25 mM glucose) (Cytiva SH30243.LS) supplemented with 10% fetal bovine serum (FBS) and antibiotics (A8943.0100, Panreac Applichem), as described previously [19]. As standard procedure, all cell lines were periodically tested for mycoplasma contamination.

Sodium dichloroacetate (DCA) (347795) and 2-deoxyglucose (2-DG) (D8375) was purchased from Sigma-Aldrich. DCA and 2-DG were dissolved in DMEM media, before being added to the cells in culture.

### Cell proliferation assays

Cell proliferation was evaluated by counting viable cells performing a Trypan blue (Sigma-Aldrich) exclusion assay. Cells were seeded at 2 × 10^5^ cells/60 mm dishes, 48 h later the cells floating in the medium were collected and the adherent cells were trypsinized, cells were pelleted and resuspended in a culture medium, mixed with a 0.4% Trypan blue/PBS solution (1:1), loaded on a hemocytometer, and Trypan blue-negative (viable cells) and Trypan blue-positive cells (dead cells) were counted.

### Protein extraction, quantification and Western blotting

After being washed with cold phosphate buffer saline (PBS), cells were lysed using CST buffer supplemented with phosphatase and protease inhibitors [19]. Proteins present at the lysates were quantified using a Bradford assay (J61522-K2 Alfa Aesar) or a BCA assay (Pierce, Rockford, IL, USA). Protein samples (30 µg) were mixed together with Laemmli Buffer and heated at 95 °C for 5 min, separated in 8% or 10% SDS/PAGE gels and electroblotted to polyvinylidene fluoride (PVDF) methanol-activated membrane (GE Healthcare Life Sciences). The membranes were blocked in Tris-buffered saline with 0.1% Tween® 20 detergent (TBS-T) supplemented with 5% non-fat milk, for at least 1 h in room temperature, before labeled with primary antibodies diluted at the indicated concentrations in TBS-T 5% bovine serum albumin (BSA) overnight at 4 °C with gentle shaking. After washing in TBS-T buffer the membranes were incubated for at least 1 h in respective secondary antibodies at the appropriate dilutions. The protein bands were visualized using the iBright 1500 Imaging System (Invitrogen, Thermo Fisher Scientific). Densitometric analysis of the target protein and respective loading control bands was performed using Fiji/ImageJ software (v1.53c). Digital images, originally acquired as black bands on a clear background, were converted to 8-bit grayscale and inverted to display white, emitting bands against a dark background. For every blot, a uniform region of interest was defined using the rectangular selection tool to encapsulate individual protein bands. The mean value for each glowing band and adjacent dark region devoid of signal (representing the local membrane background) were measured using the “Analyze> Measure” function. To isolate the true signal intensity, the calculated background mean value was subtracted from the mean value of its respective protein band. Finally, target protein levels were normalized against the corresponding loading control intensities to determine relative protein expression.

The following primary antibodies were used: total PDHA1 (ab168379), total OXPHOS human WB antibody cocktail (ab110411), and Citrate synthase (ab96600) from Abcam; and TRIB2 (D8P2X, #13533), LDHA (C4B5, #3582S), PKM2 (D78A4, #4053S), Hexokinase I (C35C4), Hexokinase II (C64G5) from Cell Signaling Technology; PDKA (12949-1-AP), Phospho-PDH E1 Alpha (Ser300) (29583-1-AP) and Phospho-PDH E1 Alpha (Ser293) (80688-2-RR) from Proteintech. Secondary antibodies were conjugated with horseradish peroxidase (GE Healthcare Life Sciences) and visualized by the ECL detection solution. Whenever possible based on the molecular size of the proteins, membranes were re-stained with different antibodies, including antibodies for protein loading control (rabbit anti-GAPDH, glyceraldehyde 3-phosphate dehydrogenase (sc-25778, Santa Cruz Biotechnology) and mouse anti-alpha Tubulin (T9026, Sigma-Aldrich). Secondary antibodies were ECL Mouse IgG, horseradish peroxidase (HRP)-linked whole Ab (from sheep) (NA931) and ECL Rabbit IgG, HRP-linked whole Ab (from donkey) (NA934).

### Lactate and glucose measurements

For glucose and lactate level measurements, melanoma cells were plated in 60 mm dishes at a final density of 5x10^5^ cells and incubated at 37ºC for 24 h. Cells were then placed in DMEM without glucose and glutamine for 1 h. After the fasting period, cells were placed back in complete DMEM (time 0) for additional 4 h (time 4). An aliquot of culture media was collected both at time 0 and time 4 and stored at −80°C until analysis. Cells were detached with PBS and stored at −80°C for protein extraction. Glucose and lactate levels present in the conditioned medium in culture were quantified using standard enzymatic techniques, subtracted to the initial levels (time 0), and normalized by protein amount (obtained and quantified as described above), as previously described [20].

### Citrate synthase activity assay

Citrate synthase (CS) activity was determined using 0.2 mM acetyl-CoA, 0.5 mM oxaloacetate (OAA), and 0.1 mM 5,5’-dithiobis(2-nitrobenzoic acid) DTNB in 100 mM Tris-HCl (pH 7.5) [21]. The reaction was followed spectrophotometrically at 412 nm at room temperature. Enzymatic activity was normalized by total protein amount.

### RNA extraction and gene expression analysis

For gene expression analysis, cells were washed with cold PBS, pelleted and stored at −20 °C until further processing. Cellular RNA was extracted using the E.Z.N.A. Total RNA Kit I, including DNase digestion (R6834-02, VWR). Total RNA concentration was estimated using a Nanodrop Spectrophotometer and 2 µg were reversely transcribed using the NZY First-Strand cDNA Synthesis Kit (MB12502, Nzytech) according to manufacturer instructions.

### Bioenergetics assay

The oxidative phosphorylation and glycolytic profile of UACC-62 melanoma cells were obtained by measuring the oxygen consumption rate (OCR) and extracellular acidification ratio (ECAR), on a Seahorse XF^e^96 Extracellular Flux Analyzer (Agilent Technologies, Germany). For OCR and ECAR, cells were seeded 12 000 cells/per well onto an XF96 Cell Culture Microplate and allowed to adhere for 24 h at 37ºC. A volume of 200 μL of calibration buffer was placed into each well of the sensor hydration microplate and the sensor cartridge placed onto the microplate. The plate was incubated with immersed sensors in a non-CO^2^ incubator overnight at 37°C. For OCR analysis, assay medium (DMEM D5030, Sigma) was supplemented with 4,5 g/L glucose, 4.0 mM L-glutamine and 1 mM sodium pyruvate, pH adjusted to 7.4, at 37°C; whilst for ECAR no glucose was added. The following day the cell medium was aspirated and gently each well was washed with assay medium at 37 °C. Then, 180 µL of assay medium at 37°C was added to each well and the plate placed into a non-CO^2^ incubator at 37°C for 1 h to reach equilibrium. To measure OCR, four compounds were added into successive injection ports, with the following final concentrations: A – 1 μM oligomycin; B – 1 μM carbonyl cyanide 4-(trifluoromethoxy)phenylhydrazone (FCCP); and C – 1 μM rotenone plus 1 μM antimycin A. For the ECAR analysis the following compounds were injected into the delivery ports: A – saturated concentration of glucose (10 mM), B – 1 μM oligomycin and C – 50 mM 2-deoxyglucose (2-DG). The protocol consists of three measurement cycles before the first injection and three measurement cycles after each port injection. Each measurement cycle consisted of 30 s mixing and 3 min of measurement. After the assay was completed, cells were fixed by adding 50 μL of 60% trichloroacetic acid and stored for 24 h at 4ºC. Cell mass was quantified by Sulforhodamine B assay and used to normalize individual wells. All the results analyses were performed using the Software Wave Desktop Version 2.2.

### Determination of intracellular superoxide levels

Intracellular superoxide levels were assessed using hydroethidine fluorescence. Cells were seeded on 10-mm glass coverslips in 24-well plates at a density of 1.5 × 10⁴ cells/well. After 24 h, the medium was replaced and cells were treated with DCA (25 mM), 2-DG (5 mM), or left untreated for an additional 48 h.

Cells were then incubated with 10 μM hydroethidine for 1 h at 37 °C in a humidified atmosphere containing 5% CO_2_, washed with PBS, and fixed with 4% paraformaldehyde containing 5 μg/mL Hoechst 33342 for 15 min at room temperature. Samples were mounted on glass slides using ProLong mounting medium. Fluorescence images were acquired 24 h later at 20 × magnification using a Nikon Eclipse 90i fluorescence microscope (Nikon) with excitation at 546 nm. Fluorescence signal was quantified using ImageJ software by measuring the integrated density of each image. The obtained values were normalized to the number of cells in the corresponding field, determined by counting Hoechst-stained nuclei. Integrated density per cell was used as an estimate of intracellular superoxide levels.

### Statistical analysis

Statistical analysis was performed using GraphPad Prism software (version 8.4.0, San Diego, CA, USA). ANOVA test was used to evaluate statistical differences between the WT and TRIB2-KO cells. When indicated, multiple comparisons were performed. *P* < 0.05 was considered statistically significant.

## Results

### TRIB2 depletion reduces melanoma cell proliferation and alters the response to metabolic inhibitors

High endogenous TRIB2 protein levels were formerly detected in melanoma cells [15]. We previously developed isogenic TRIB2 knock out (KO) cells in UACC-62 melanoma cell line [16]. To investigate whether TRIB2 influences melanoma cell proliferation and the response to metabolic stress, we quantified viable cells by trypan blue exclusion and cell counting. Under basal conditions, both TRIB2-KO clones (#28 and #31) displayed significantly reduced proliferation compared with WT cells after 48 h (Fig 1A), confirming the contribution of TRIB2 to melanoma cell growth.

**Fig 1:**
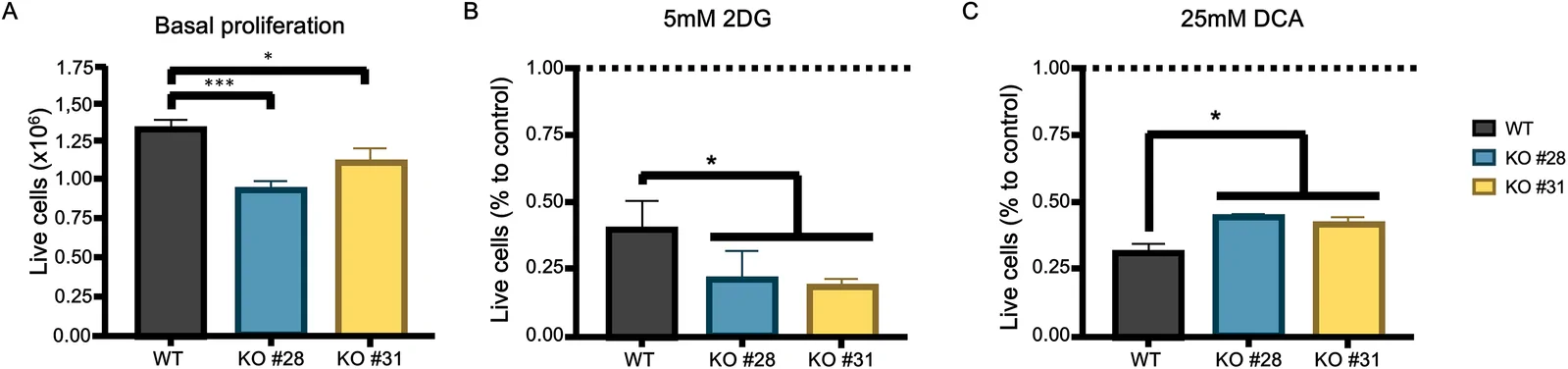
TRIB2 depletion alters melanoma cell proliferation and sensitivity to metabolic inhibitors. Cell proliferation in WT and TRIB2-KO clones #28 and #31 determined using a trypan blue exclusion assay after 48 h. (**A**) Basal proliferation represented by number of live cells, (**B**) 5 mM 2-deoxy-D-glucose (2-DG) and (**C**) 25 mM dichloroacetate (DCA), expressed as percentage relative to the corresponding untreated control. Data represent mean ± SEM from three independent experiments performed in triplicate. Statistical significance was determined using one-way ANOVA followed by Tukey’s multiple-comparison test. Significant differences are indicated as *p < 0.05, **p < 0.01, and ***p < 0.001.

We next examined cell proliferation following glycolysis inhibition using the hexokinase inhibitor 2-deoxyglucose (2-DG) [22]. After 48 h of treatment, TRIB2-KO cells showed a significantly greater reduction in proliferation than WT cells (Fig 1B), indicating an increased sensitivity to glycolytic inhibition in the absence of TRIB2.

Finally, when exposed to dichloroacetic acid (DCA), which selectively inhibits pyruvate dehydrogenase kinases (PDKs) and consequently impairs glycolysis favoring mitochondrial oxidative metabolism [23,24], WT cells exhibited a significantly greater reduction in proliferation than TRIB2-KO cells after 48 h treatment with 25 mM DCA (Fig 1C). Thus, while TRIB2-deficient cells were more susceptible to glycolytic inhibition by 2-DG, TRIB2-expressing cells were more sensitive to DCA treatment. Together, these results indicate that TRIB2 expression influences both basal melanoma cell proliferation and the cellular response to metabolic perturbation, suggesting a role for TRIB2 in the regulation of metabolic adaptation.

### Cells with high TRIB2 levels show preferential glycolytic metabolism

Given the differential response of WT and TRIB2-KO cells to metabolic inhibitors, we next investigated whether TRIB2 expression was associated with alterations in cellular metabolism. We found both increased glucose consumption and lactate production in WT cells compared to TRIB2-KO cells, cultured in high glucose media (Fig 2A, B).

**Fig 2:**
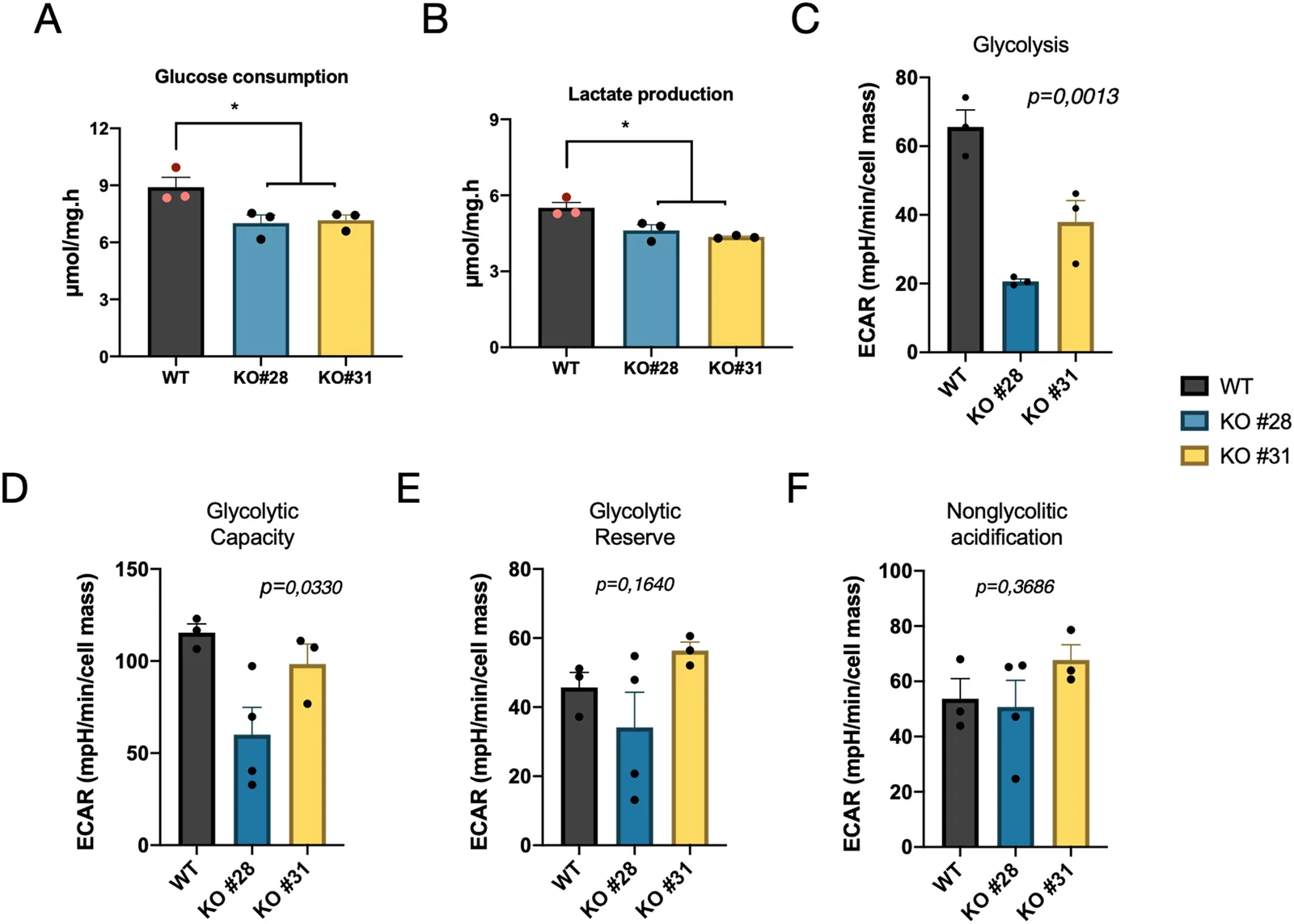
Cells with high TRIB2 levels show preferential glycolytic metabolism. (**A**) Glucose consumption and (**B**) lactate secretion, represented as µmol/mg.h, from parental (WT) and 2 independent clones of TRIB2-KO (#28 and #31) UACC-62 cells, cultured for 4 h in high glucose media after a 1-hour fasting period (DMEM without glucose), obtained from spectrometry-based enzymatic assays (n = 3 independent assays performed in triplicate). (**C-F**) Individual parameters of glycolysis based on the extracellular acidification rate (ECAR) in WT and TRIB2-KO #28 and #31 cells, evaluated through the Seahorse bioanalyzer (n = 3-4 assays performed with 5 replicates). Values are mean + - standard error mean (SEM). Tukey’s multiple comparisons test *p* values indicated as * (*p* < 0.05) (**A, B**) or ANOVA *p* values shown (**C-F**).

To determine TRIB2-induced global changes in metabolic features, we evaluated the oxygen consumption rates (OCR), in parallel with extracellular acidification rate (ECAR), which allow the real-time analysis of both oxidative and glycolytic metabolism in live cells, respectively. Functional assays revealed decreased glycolysis and glycolytic capacity in TRIB2-KO cells, compared to WT cells (Figs 2C, D, S1A). Notably, these differences did not extend to their glycolytic reserve or levels of non-glycolytic acidification, as statistical significance was absent (Fig 2E, F). Together, these findings demonstrate that TRIB2 expression is associated with enhanced glycolytic activity, as evidenced by increased glucose consumption, lactate secretion and ECAR measurements.

### TRIB2 expression does not alter mitochondrial respiration

Given the reduced glycolytic phenotype observed in TRIB2-KO cells, we next investigated whether these metabolic changes were accompanied by alterations in mitochondrial respiration and function. However, we found no significant differences in any of the measurements performed based on the OCR, namely basal respiration, maximal respiratory capacity, ATP linked respiration, nor in spare respiratory capacity and proton leak, between WT and TRIB2-KO cells (Figs 3A-E; S1B).

**Fig 3:**
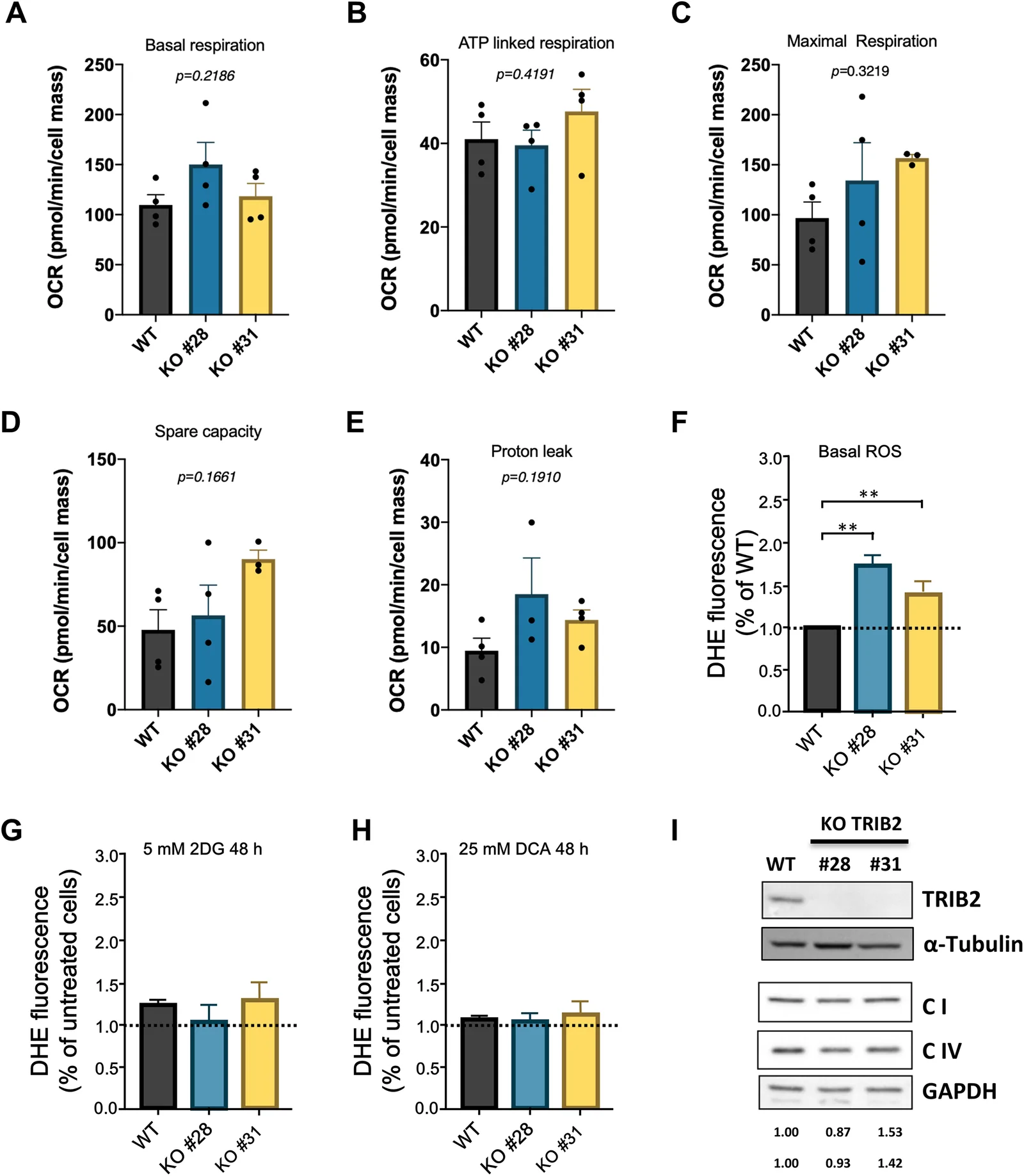
TRIB2 depletion does not impair mitochondrial respiratory function. (**A-E**) Individual parameters of mitochondrial function based on the oxygen consumption rate (OCR) in WT and TRIB2-KO #28 and #31 cells, evaluated through the Seahorse bioanalyzer (n = 3-4 assays performed with 5 replicates). (**F-H**) Intracellular superoxide levels measured by dihydroethidium (DHE) fluorescence in WT and TRIB2-KO #28 and #31 cells under basal conditions (**F**), following treatment with 5 mM 2-deoxy-D-glucose (2-DG) for 48 h (**G**), or 25 mM dichloroacetate (DCA) for 48 h (**H**) (n = 3 independent experiments). Values are mean + - standard error mean (SEM). ANOVA test *p* values are indicated. (**I**) Mitochondrial proteins levels in whole cell extracts from parental (WT) and TRIB2-KO (clone #28 and #31) UACC-62 melanoma cell line, using GAPDH as loading control.

Although not statistically significant, both TRIB2-KO clones showed a trend towards increased proton leak compared with WT cells (Fig 3E). Since alterations in proton leak have been associated with changes in mitochondrial redox homeostasis, we next evaluated intracellular superoxide levels using dihydroethidium (DHE) fluorescence. Under basal conditions, both TRIB2-KO clones exhibited significantly higher superoxide levels than WT cells (Fig 3F). In contrast, no significant differences were observed following treatment with either 2-deoxy-D-glucose (2-DG) or dichloroacetate (DCA) (Fig 3G, H), indicating that TRIB2 depletion is associated with increased basal intracellular superoxide levels, whereas no significant differences were detected following treatment with either 2-DG or DCA.

To further assess whether TRIB2 depletion affects mitochondrial content, we analyzed representative mitochondrial markers. Protein levels of OXPHOS complex II (UQCRC2), and complex IV (COX II) were comparable between WT and TRIB2-KO cells (Fig 3I), supporting the notion that loss of TRIB2 does not induce major alterations in mitochondrial abundance or respiratory capacity.

### TRIB2 depletion selectively alters PDK4 expression and citrate synthase activity

To identify molecular alterations associated with the metabolic phenotype observed in TRIB2-deficient cells, we analyzed the expression of multiple genes and proteins involved in glycolysis, pyruvate metabolism and the tricarboxylic acid (TCA) cycle.

Several transcriptional changes were detected in TRIB2-KO cells, including reduced expression of HK1 and citrate synthase (CS), together with increased expression of PFKL, ALDOC, GYS1, IDH1 and PDK1 (S2 Fig). In contrast, the expression of GLUT1, PGM1, PKM, GPI, PGC1A, CPT1A and GSK3B was not significantly altered (S2 Fig).

Among the genes analyzed, PDK4 showed the most consistent reduction, displaying significantly lower mRNA levels in both TRIB2-KO clones compared with WT cells (Fig 4A). This decrease was also observed at the protein level. In contrast, although PDK1 transcript levels were increased in TRIB2-KO cells, no differences in PDK1 protein abundance were detected between WT and KO cells (Fig 4A, B).

**Fig 4:**
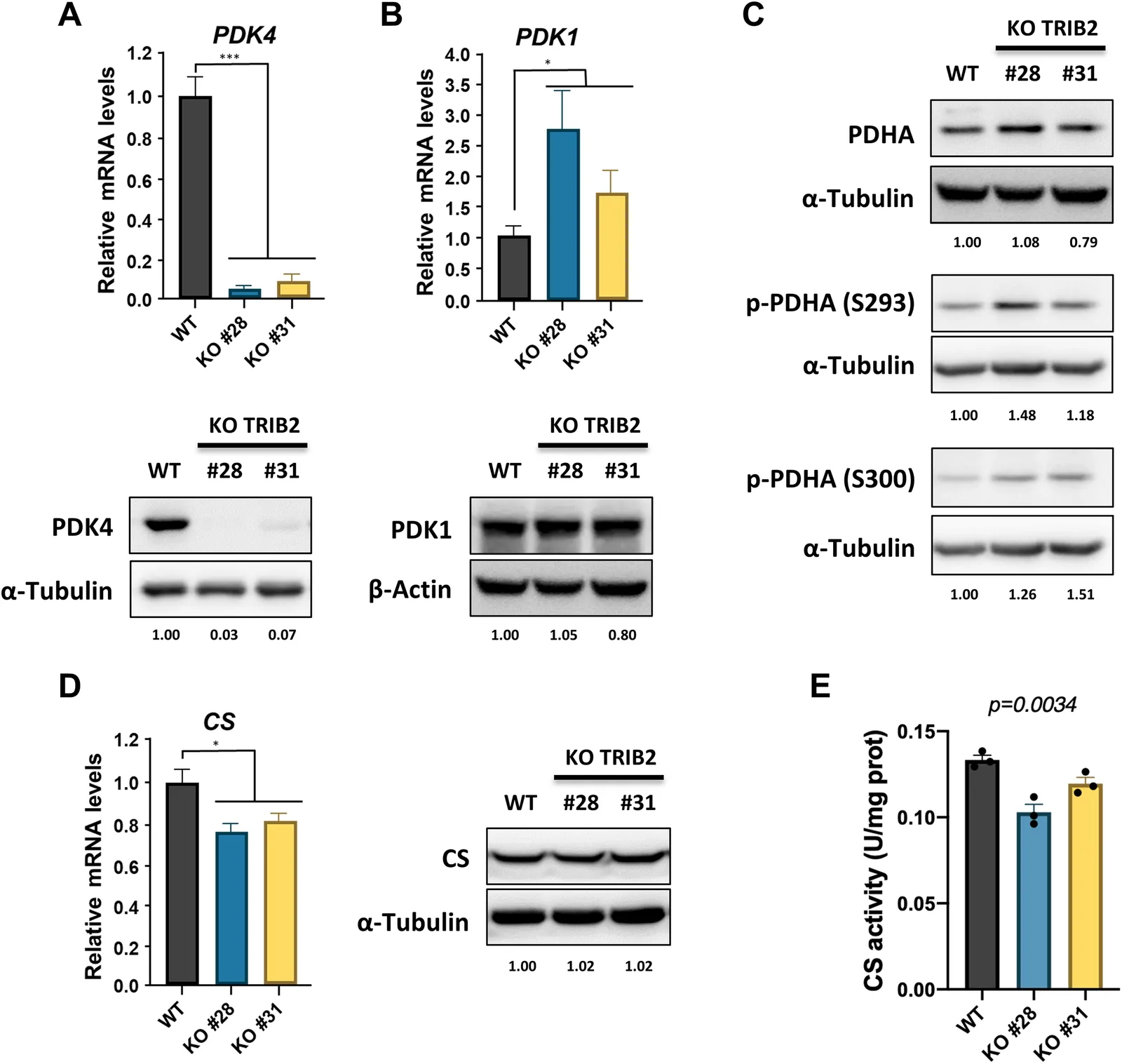
TRIB2 depletion reduces PDK4 expression and citrate synthase activity. (**A**) Relative PDK4 mRNA expression, normalized to GAPDH, and representative immunoblot of PDK4 protein levels in parental (WT) and TRIB2-KO (#28 and #31) UACC-62 melanoma cells. (**B**) Relative PDK1 mRNA expression, normalized to GAPDH, and representative immunoblot of PDK1 protein levels. (**C**) Representative immunoblots showing total pyruvate dehydrogenase E1 alpha subunit (PDHA) and phosphorylated PDHA at Ser293 and Ser300. (**D**) Relative citrate synthase (CS) mRNA expression, normalized to GAPDH, and representative immunoblot of CS protein levels. (**E**) Citrate synthase enzymatic activity in WT and TRIB2-KO cells.

To determine whether these changes affected the pyruvate dehydrogenase complex, we assessed total PDHA protein levels together with phosphorylation at Ser293 and Ser300. No consistent differences in total PDHA abundance were observed between WT and TRIB2-KO cells. In contrast, phosphorylation of PDHA at Ser293 and Ser300 appeared higher in both TRIB2-KO clones (Fig 4C).

We next evaluated citrate synthase, a key enzyme of the TCA cycle. CS mRNA levels were reduced in TRIB2-KO cells, whereas protein abundance remained comparable to WT cells (Fig 4D). However, citrate synthase enzymatic activity was significantly decreased in both TRIB2-KO clones (Fig 4E).

Additional proteins involved in glycolysis, including HK1, HK2, LDHA and PKM2, showed no detectable differences between WT and TRIB2-KO cells (S3 Fig).

## Discussion

TRIB2, recognized as an oncogene which interacts with proteins involved in cell survival and proliferation [25], has previously demonstrated a distinct role in fueling the growth and survival of melanoma cells [15]. In the present study, we extend these observations by demonstrating that TRIB2 also contributes to the metabolic phenotype of melanoma cells. TRIB2 depletion reduced basal cell proliferation together with glucose consumption, lactate production and extracellular acidification rate (ECAR), indicating that TRIB2 expression favours a glycolytic phenotype. Functionally, these metabolic changes were accompanied by increased sensitivity of TRIB2-deficient cells to glycolytic inhibition by 2-deoxy-D-glucose, whereas TRIB2-expressing cells were more sensitive to dichloroacetate. Together, these findings suggest that TRIB2 supports the metabolic flexibility required to sustain melanoma cell proliferation.

Melanoma exhibits remarkable metabolic plasticity, allowing tumour cells to dynamically adapt their bioenergetic state according to nutrient availability, oncogenic signalling and therapeutic pressure [2,26]. Rather than relying exclusively on aerobic glycolysis, melanoma cells can exploit both glycolytic and oxidative pathways, and alterations in this balance have been associated with tumour progression and therapeutic response [2,27]. In agreement with this concept, our data identify TRIB2 as a novel contributor to melanoma metabolic adaptation by promoting glucose utilization and lactate production.

To date, only one study has directly linked TRIB2 to glucose metabolism. Liu et al. reported that TRIB2 promotes aerobic glycolysis in lung cancer cells through interaction with PKM2, increasing both glucose uptake and lactate secretion [18]. Our results independently support a pro-glycolytic role for TRIB2, as TRIB2-expressing melanoma cells also exhibited higher glucose consumption and lactate production than TRIB2-KO cells. However, unlike Liu et al., we did not detect changes in PKM2 protein abundance, nor in LDHA, HK1 or HK2 protein levels. These differences most likely reflect tumour-specific metabolic regulation and suggest that, in melanoma, TRIB2 promotes glycolysis through mechanisms distinct from the PKM2-dependent pathway described in lung cancer.

Interestingly, despite the clear reduction in glycolytic activity, TRIB2 depletion did not result in a compensatory increase in mitochondrial respiration. Neither basal nor maximal oxygen consumption, ATP-linked respiration nor spare respiratory capacity differed between WT and TRIB2-KO cells, indicating that loss of TRIB2 does not simply redirect glucose-derived carbon towards oxidative phosphorylation. This observation is consistent with the current view that metabolic adaptation in melanoma is not governed by a binary switch between glycolysis and oxidative phosphorylation, but rather by coordinated regulation of multiple interconnected metabolic pathways [2,27]. Indeed, previous studies have shown that glycolytic activity and mitochondrial respiration frequently coexist in melanoma cells and may be regulated independently according to the genetic and metabolic context [28,29].

Although the increase in proton leak did not reach statistical significance, both TRIB2-KO clones consistently displayed higher basal superoxide levels. Proton leak and mitochondrial ROS production are closely interconnected, and modest alterations in proton conductance can modify mitochondrial redox homeostasis depending on the metabolic state rather than following a simple linear relationship [30]. Therefore, our data suggest that TRIB2 depletion primarily affects basal cellular redox homeostasis without inducing major alterations in respiratory function.

To further investigate the molecular basis underlying the metabolic phenotype associated with TRIB2 depletion, we analysed the expression of several genes and proteins involved in glycolysis, pyruvate metabolism and the tricarboxylic acid cycle. Among all the molecules examined, the most consistent molecular alteration identified in TRIB2-deficient cells was the reduction of PDK4, observed at both the mRNA and protein levels. PDK4 is one of the four pyruvate dehydrogenase kinases that regulate the activity of the pyruvate dehydrogenase complex (PDC), thereby controlling the entry of pyruvate into the tricarboxylic acid cycle. However, the contribution of individual PDK isoforms is highly context-dependent, and substantial functional redundancy exists between family members [31–33].

Despite the marked reduction in PDK4 expression, PDHA phosphorylation was modestly increased in both knockout clones. Since PDC activity is determined by the integrated action of all PDK isoforms together with pyruvate dehydrogenase phosphatases, rather than by the abundance of a single kinase [31,32], this observation may reflect compensatory regulation by other PDK family members.

Our observations are also consistent with previous findings indicating that the metabolic consequences of PDK4 modulation cannot always be explained solely by changes in PDH phosphorylation. Tiersma et al. demonstrated that selective downregulation of individual PDK isoforms, including PDK4, did not produce proportional changes in PDH phosphorylation in melanoma cells, highlighting the robustness and functional redundancy of PDC regulation [34].

Beyond its canonical role in regulating PDH, increasing evidence indicates that PDK4 exerts additional biological functions. In KRAS-driven tumour models, PDK4 depletion impaired cell proliferation and altered KRAS signalling independently of PDH activity, supporting PDH-independent roles in tumour cell metabolism and proliferation [35]. Likewise, PDK4 has been shown to directly phosphorylate septin 2 (SEPT2), thereby regulating DRP1-dependent mitochondrial dynamics independently of PDH phosphorylation [36]. In addition, PDK4 has been implicated in the modulation of ERK, SRC and JNK signalling pathways in bladder cancer cells, further supporting functions unrelated to its classical control of pyruvate oxidation [37].

Although our study does not address these mechanisms directly, these observations provide a plausible framework to explain why reduced PDK4 expression is associated with a clear metabolic phenotype despite preserved regulation of the pyruvate dehydrogenase complex. Together, these findings suggest that TRIB2 regulates melanoma metabolism, at least in part, through modulation of PDK4. However, the downstream consequences of PDK4 depletion are likely to extend beyond the canonical regulation of PDH phosphorylation.

Interestingly, analysis of publicly available Cancer Dependency Map (DepMap) datasets from the Cancer Cell Line Encyclopedia (CCLE) [38] further supports the biological relevance of this relationship, as TRIB2 and PDK4 expression positively correlate across several tumour types, including melanoma, glioma, acute myeloid leukaemia, pancreatic adenocarcinoma and endometrial carcinoma (S4–S5 Figs). Although these correlations do not establish a causal relationship, they indicate that the association between TRIB2 and PDK4 extends beyond the UACC-62 melanoma model and supports the potential biological relevance of the TRIB2–PDK4 axis across multiple tumour types.

A second major molecular alteration was the reduction in citrate synthase activity observed in TRIB2-KO cells. Although CS transcript levels were decreased and protein abundance remained unchanged, enzymatic activity was significantly reduced. Citrate synthase catalyses the first committed step of the tricarboxylic acid cycle and its activity is widely used as a surrogate marker of mitochondrial content and oxidative capacity [39]. This dissociation between protein abundance and enzymatic activity suggests regulation beyond protein expression, potentially through post-translational or metabolic mechanisms that were not addressed in the present work. Accordingly, the reduced citrate synthase activity most likely reflects functional metabolic remodelling rather than decreased mitochondrial content, consistent with the preserved respiratory capacity and unchanged abundance of representative mitochondrial proteins observed in our study.

Taken together, the two most consistent molecular alterations identified in TRIB2-deficient cells—reduced PDK4 expression and decreased citrate synthase activity— both converge on altered regulation of mitochondrial carbon metabolism despite the preservation of mitochondrial respiratory function.

Our functional studies further indicate that TRIB2 expression influences the response of melanoma cells to metabolic therapies. TRIB2-deficient cells were more sensitive to glycolytic inhibition by 2-deoxy-D-glucose, whereas TRIB2-expressing cells displayed greater sensitivity to dichloroacetate. These findings reinforce the concept that metabolic dependencies differ according to the metabolic phenotype of tumour cells and suggest that TRIB2 expression may contribute to defining distinct metabolic vulnerabilities. Although these observations require validation in additional melanoma models and in vivo studies, they support the rationale for considering TRIB2 as a potential biomarker to guide metabolism-targeted therapeutic strategies.

Collectively, our findings indicate that TRIB2 depletion does not simply trigger a metabolic shift from glycolysis towards oxidative metabolism. Instead, loss of TRIB2 promotes a broader metabolic rewiring characterized by reduced glycolytic activity together with selective alterations in mitochondrial carbon metabolism, while preserving overall mitochondrial respiratory capacity. This metabolic phenotype is supported by the coordinated reduction in PDK4 expression and citrate synthase activity, identifying the TRIB2–PDK4 axis as a previously unrecognized component of metabolic regulation in melanoma. Although the molecular mechanisms linking TRIB2 to PDK4 remain to be elucidated, our findings provide a framework for future studies aimed at defining how TRIB2 controls metabolic adaptation in melanoma and whether this regulatory axis represents a therapeutic vulnerability in other tumor types.

## Supporting information

S1 Fig Seahorse analysis. (A) Agilent Seahorse XF96 analysis of glycolytic function based on the extracellular acidification rate (ECAR) in WT and TRIB2-KO #28 and #31 cells, in basal media without glucose, and in response to glucose (Gluc), oligomycin (Oligo) and 2-deoxy-glucose (2-DG). (B) Agilent Seahorse XF96 analysis of mitochondrial respiration based on the oxygen consumption rate (OCR) in WT and TRIB2-KO #28 and #31 cells, in basal media and in response to oligomycin, carbonyl cyanide-4 (trifluoromethoxy) phenylhydrazone (FCCP) and a mix of rotenone (Rot) and antimycin A (AA). Values are mean + - standard error mean (SEM).
(TIF)

S2 Fig TRIB2 depletion does not alter the expression of additional glycolytic or mitochondrial metabolic genes. (A–G) Relative mRNA expression of solute carrier family 2 member 1 (SLC2A1, GLUT1), phosphoglucomutase 1 (PGM1), pyruvate kinase M (PKM), glucose-6-phosphate isomerase (GPI), peroxisome proliferator-activated receptor gamma coactivator 1-alpha (PPARGC1A, PGC1α), carnitine palmitoyltransferase 1A (CPT1A) and glycogen synthase kinase-3 beta (GSK3B), normalized to GAPDH, in parental (WT) and two independent TRIB2-KO clones (#28 and #31) UACC-62 cells (n = 3 independent assays). Values are mean ± SEM of fold change relative to WT. One-way ANOVA was performed and p-values are indicated in each graph.
(TIF)

S3 Fig Depletion of TRIB2 does not alter the levels of proteins involved in metabolism. Protein levels of HK1 and HK2 (A), and LDHA and PKM2 (B), in whole cell extracts from parental (WT) and TRIB2-KO (clone #28 and #31) UACC-62 melanoma cell line, using Tubulin as loading control.
(TIF)

S4 Fig Correlation between TRIB2 and PDK4 mRNA expression across different tumour types. Scatter plots showing the correlation between TRIB2 and PDK4 mRNA expression (RNA-Seq TPM) in pancreatic adenocarcinoma, acute myeloid leukaemia, melanoma, glioma and endometrial carcinoma. Pearson’s and Spearman’s correlation coefficients and corresponding *P* values are indicated in each panel. Data were obtained from the Cancer Dependency Map (DepMap) portal, which integrates molecular characterization of cancer cell lines from the Cancer Cell Line Encyclopedia (CCLE). Figure generated using the DepMap data visualization tool. Data source: Arafeh R, Shibue T, Dempster JM, Hahn WC, Vazquez F. *The present and future of the Cancer Dependency Map*. Nat Rev Cancer. 2025;25(1):59–73. https://doi.org/10.1038/s41568-024-00763-x.
(TIF)

S5 Fig Full membranes of western blots. (TIF)

S1 File Raw images. (PDF)

## References

[1] Hanahan D, Weinberg RA. Hallmarks of cancer: The next generation. Cell. 2011;144(5):646–74. doi: 10.1016/j.cell.2011.02.013 21376230

[2] Ruocco MR, Avagliano A, Granato G, Vigliar E, Masone S, Montagnani S, et al. Metabolic flexibility in melanoma: A potential therapeutic target. Semin Cancer Biol. 2019;59:187–207. doi: 10.1016/j.semcancer.2019.07.016 31362075

[3] Furue M, Ito T, Wada N, Wada M, Kadono T, Uchi H. Melanoma and immune checkpoint inhibitors. Curr Oncol Rep. 2018;20(3):29. 10.1007/s11912-018-0676-z 29569208

[4] Luke JJ, Flaherty KT, Ribas A, Long GV. Targeted agents and immunotherapies: Optimizing outcomes in melanoma. Nat Rev Clin Oncol. 2017;14(8):463–82. doi: 10.1038/nrclinonc.2017.43 28374786

[5] Yokoyama T, Nakamura T. Tribbles in disease: Signaling pathways important for cellular function and neoplastic transformation. Cancer Sci. 2011;102(6):1115–22. doi: 10.1111/j.1349-7006.2011.01914.x 21338441

[6] O’Connor C, Yalla K, Salomé M, Moka HA, Castañeda EG, Eyers PA, et al. Trib2 expression in granulocyte-monocyte progenitors drives a highly drug resistant acute myeloid leukaemia linked to elevated Bcl2. Oncotarget. 2018;9(19):14977–92. doi: 10.18632/oncotarget.24525 29599919 PMC5871090

[7] Ma X, Zhou X, Qu H, Ma Y, Yue Z, Shang W, et al. TRIB2 knockdown as a regulator of chemotherapy resistance and proliferation via the ERK/STAT3 signaling pathway in human chronic myelogenous leukemia K562/ADM cells. Oncol Rep. 2018;39(4):1910–8. doi: 10.3892/or.2018.6249 29436678

[8] Liang Y, Yu D, Perez-Soler R, Klostergaard J, Zou Y. TRIB2 contributes to cisplatin resistance in small cell lung cancer. Oncotarget. 2017;8(65):109596–608. doi: 10.18632/oncotarget.22741 29312632 PMC5752545

[9] Wang J, Zuo J, Wahafu A, Wang M, Li R-C, Xie W-F. Combined elevation of TRIB2 and MAP3K1 indicates poor prognosis and chemoresistance to temozolomide in glioblastoma. CNS Neurosci Ther. 2020;26(3):297–308. doi: 10.1111/cns.13197 31318172 PMC7053231

[10] Ferreira BI, Santos B, Link W, De Sousa-Coelho AL. Tribbles pseudokinases in colorectal cancer. Cancers. 2021;13(11). 10.3390/cancers13112825 PMC8201230 34198908

[11] Fernandes MT, Yassuda V, Bragança J, Link W, Ferreira BI, De Sousa-Coelho AL. Tribbles gene expression profiles in colorectal cancer. Gastrointest Disord. 2021;3(4):218–36.

[12] Liu C, Lu Z, Liu H, Zhuang S, Guo P. LncRNA XIST promotes the progression of laryngeal squamous cell carcinoma via sponging miR-125b-5p to modulate TRIB2. Biosci Rep. 2020;40(4):BSR20193172. doi: 10.1042/BSR20193172 32149330 PMC7146034

[13] Wei G, Lu T, Shen J, Wang J. LncRNA ZEB1-AS1 promotes pancreatic cancer progression by regulating miR-505-3p/TRIB2 axis. Biochem Biophys Res Commun. 2020;528(4):644–9. doi: 10.1016/j.bbrc.2020.05.105 32513531

[14] Monga J, Adrianto I, Rogers C, Gadgeel S, Chitale D, Alumkal JJ, et al. Tribbles 2 pseudokinase confers enzalutamide resistance in prostate cancer by promoting lineage plasticity. J Biol Chem. 2022;298(2):101556. doi: 10.1016/j.jbc.2021.101556 34973338 PMC8800106

[15] Zanella F, Renner O, García B, Callejas S, Dopazo A, Peregrina S, et al. Human TRIB2 is a repressor of FOXO that contributes to the malignant phenotype of melanoma cells. Oncogene. 2010;29(20):2973–82. doi: 10.1038/onc.2010.58 20208562

[16] Machado S, Silva A, De Sousa-Coelho AL, Duarte I, Grenho I, Santos B, et al. Harmine and piperlongumine revert TRIB2-mediated drug resistance. Cancers. 2020;12(12):3689. doi: 10.3390/cancers12123689 PMC7763856 33316942

[17] Link W. Tribbles breaking bad: TRIB2 suppresses FOXO and acts as an oncogenic protein in melanoma. Biochem Soc Trans. 2015;43(5):1085–8. doi: 10.1042/BST20150102 26517928

[18] Liu Y-R, Song D-D, Liang D-M, Li Y-J, Yan Y-F, Sun H-F, et al. Oncogenic TRIB2 interacts with and regulates PKM2 to promote aerobic glycolysis and lung cancer cell procession. Cell Death Discov. 2022;8(1):306. doi: 10.1038/s41420-022-01095-1 35790734 PMC9256704

[19] De Sousa-Coelho AL, Aureliano M, Fraqueza G, Serrão G, Gonçalves J, Sánchez-Lombardo I, et al. Decavanadate and metformin-decavanadate effects in human melanoma cells. J Inorg Biochem. 2022;235:111915. doi: 10.1016/j.jinorgbio.2022.111915 35834898

[20] Mayoral-Varo V, Calcabrini A, Sánchez-Bailón MP, Martínez-Costa ÓH, González-Páramos C, Ciordia S, et al. c-Src functionality controls self-renewal and glucose metabolism in MCF7 breast cancer stem cells. PLoS One. 2020;15(7):e0235850. doi: 10.1371/journal.pone.0235850 32673341 PMC7365443

[21] Morgunov I, Srere PA. Interaction between citrate synthase and malate dehydrogenase. J Biol Chem. 1998;273(45):29540–4. 10.1074/jbc.273.45.29540 9792662

[22] Zhang D, Li J, Wang F, Hu J, Wang S, Sun Y. 2-Deoxy-D-glucose targeting of glucose metabolism in cancer cells as a potential therapy. Cancer Lett. 2014;355(2):176–83. doi: 10.1016/j.canlet.2014.09.003 25218591

[23] Kankotia S, Stacpoole PW. Dichloroacetate and cancer: New home for an orphan drug? Biochim Biophys Acta - Rev Cancer. 2014;1846(2):617–29. 10.1016/j.bbcan.2014.08.005 25157892

[24] Tataranni T, Piccoli C. Dichloroacetate (DCA) and cancer: An overview towards clinical applications. Oxid Med Cell Longev. 2019;2019:8201079. doi: 10.1155/2019/8201079 31827705 PMC6885244

[25] Mayoral-Varo V, Jiménez L, Link W. The critical role of TRIB2 in cancer and therapy resistance. Cancers (Basel). 2021;13(11):2701. doi: 10.3390/cancers13112701 34070799 PMC8198994

[26] DeBerardinis RJ, Lum JJ, Hatzivassiliou G, Thompson CB. The biology of cancer: Metabolic reprogramming fuels cell growth and proliferation. Cell Metab. 2008;7(1):11–20. doi: 10.1016/j.cmet.2007.10.002 18177721

[27] Abildgaard C, Rizza S, Christiansen H, Schmidt S, Dahl C, Abdul-Al A, et al. Screening of metabolic modulators identifies new strategies to target metabolic reprogramming in melanoma. Sci Rep. 2021;11(1):4390. doi: 10.1038/s41598-021-83796-8 33623106 PMC7902673

[28] Haq R, Shoag J, Andreu-Perez P, Yokoyama S, Edelman H, Rowe GC, et al. Oncogenic BRAF regulates oxidative metabolism via PGC1α and MITF. Cancer Cell. 2013;23(3):302–15. doi: 10.1016/j.ccr.2013.02.003 23477830 PMC3635826

[29] Vazquez F, Lim J-H, Chim H, Bhalla K, Girnun G, Pierce K, et al. PGC1α expression defines a subset of human melanoma tumors with increased mitochondrial capacity and resistance to oxidative stress. Cancer Cell. 2013;23(3):287–301. doi: 10.1016/j.ccr.2012.11.020 23416000 PMC3708305

[30] Divakaruni AS, Brand MD. The regulation and physiology of mitochondrial proton leak. Physiology (Bethesda). 2011;26(3):192–205. doi: 10.1152/physiol.00046.2010 21670165

[31] Bowker-Kinley MM, Davis WI, Wu P, Harris RA, Popov KM. Evidence for existence of tissue-specific regulation of the mammalian pyruvate dehydrogenase complex. Biochem J. 1998;329 (Pt 1)(Pt 1):191–6. doi: 10.1042/bj3290191 9405293 PMC1219031

[32] Patel MS, Korotchkina LG. Regulation of the pyruvate dehydrogenase complex. Biochem Soc Trans. 2006;34(Pt 2):217–22. doi: 10.1042/BST20060217 16545080

[33] Atas E, Oberhuber M, Kenner L. The implications of PDK1-4 on tumor energy metabolism, aggressiveness and therapy resistance. Front Oncol. 2020;10:583217. doi: 10.3389/fonc.2020.583217 33384955 PMC7771695

[34] Tiersma JF, Evers B, Bakker BM, Jalving M, de Jong S. Pyruvate dehydrogenase kinase inhibition by dichloroacetate in melanoma cells unveils metabolic vulnerabilities. Int J Mol Sci. 2022;23(7):3745. doi: 10.3390/ijms23073745 35409102 PMC8999016

[35] Trinidad AG, Whalley N, Rowlinson R, Delpuech O, Dudley P, Rooney C, et al. Pyruvate dehydrogenase kinase 4 exhibits a novel role in the activation of mutant KRAS, regulating cell growth in lung and colorectal tumour cells. Oncogene. 2017;36(44):6164–76. doi: 10.1038/onc.2017.224 28692044 PMC5671936

[36] Thoudam T, Chanda D, Sinam IS, Kim B-G, Kim M-J, Oh CJ, et al. Noncanonical PDK4 action alters mitochondrial dynamics to affect the cellular respiratory status. Proc Natl Acad Sci U S A. 2022;119(34):e2120157119. doi: 10.1073/pnas.2120157119 35969774 PMC9407676

[37] Lee EH, Chung J-W, Sung E, Yoon BH, Jeon M, Park S, et al. Anti-metastatic effect of pyruvate dehydrogenase kinase 4 inhibition in bladder cancer via the ERK, SRC, and JNK pathways. Int J Mol Sci. 2022;23(21):13240. doi: 10.3390/ijms232113240 36362028 PMC9658024

[38] Arafeh R, Shibue T, Dempster JM, Hahn WC, Vazquez F. The present and future of the cancer dependency map. Nat Rev Cancer. 2025;25(1):59–73. doi: 10.1038/s41568-024-00763-x 39468210

[39] Larsen S, Nielsen J, Hansen CN, Nielsen LB, Wibrand F, Stride N, et al. Biomarkers of mitochondrial content in skeletal muscle of healthy young human subjects. J Physiol. 2012;590(14):3349–60. doi: 10.1113/jphysiol.2012.230185 22586215 PMC3459047

