# Supplementary material for: TRIB2 supports a high glycolytic phenotype in melanoma cells
Source: PLoS One. 2026 Nov 12;21(11):e0360503. doi: 10.1371/journal.pone.0360503 (PMC13759053; doi:10.1371/journal.pone.0360503)
Supplement: Supplementary file 1 [file pone.0360503.s006.pdf]

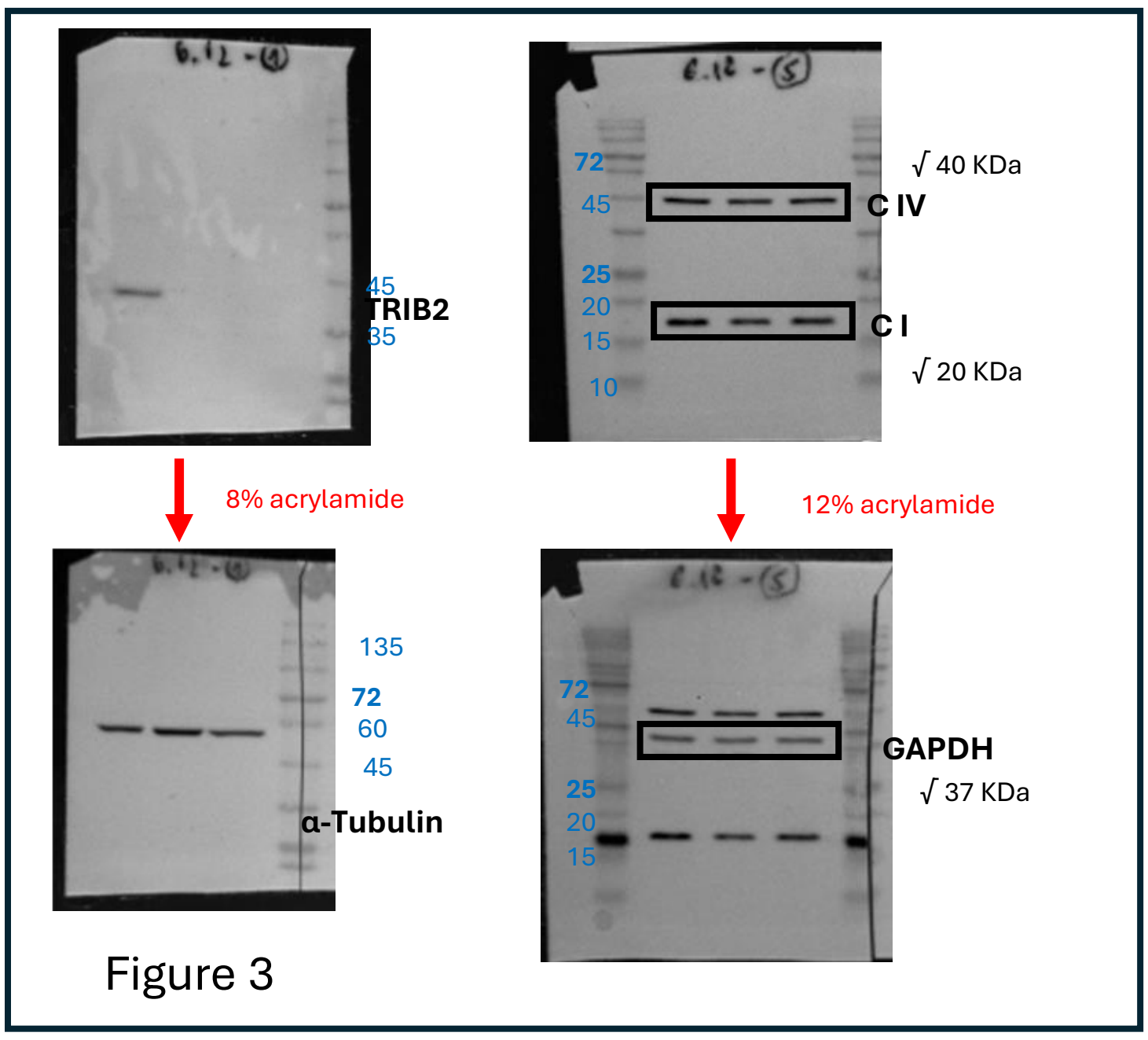

Figure 3

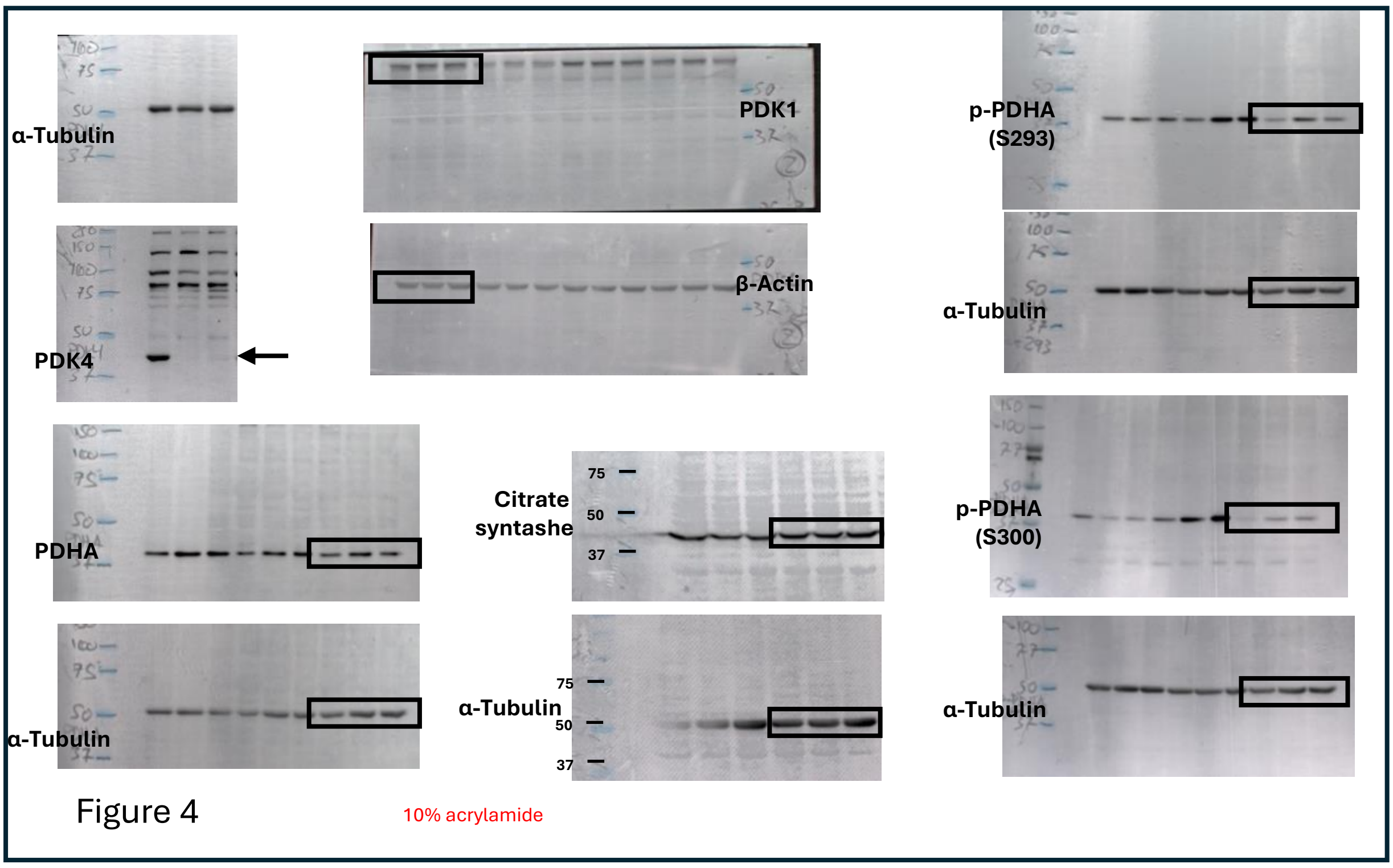

Figure 4

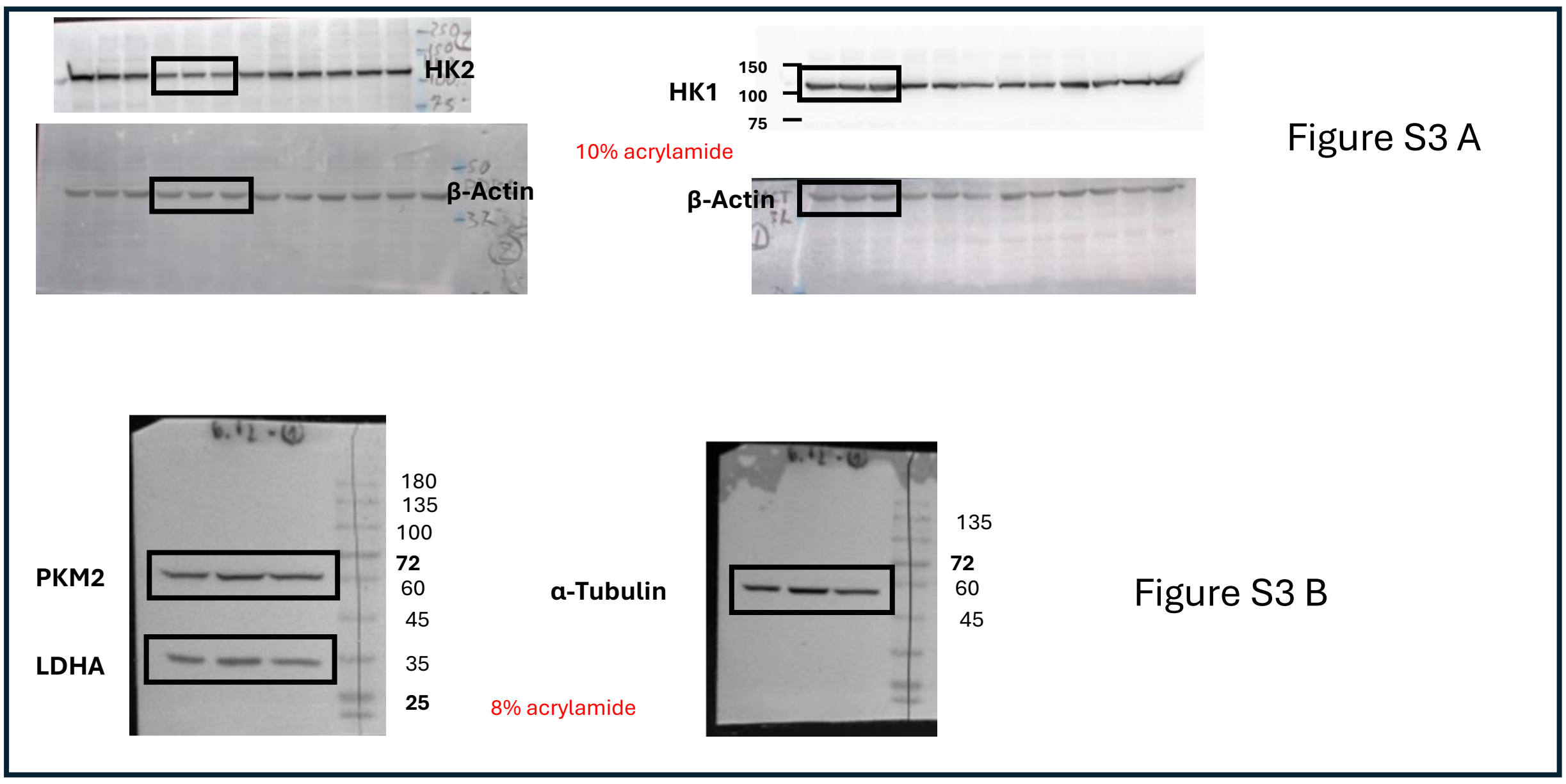

Figure S3 A

Figure S3 B
